# Selections

**Published:** 1881-08

**Authors:** 


### SELECTIONS.

### CARBUNCLE—ITS TREATMENT.

BY J. B. RICHARDSON, M. D.

"Early impressions are the most lasting," applies as forcibly to instructions in surgery as to any department of acquired knowledge. The treatment (local) of anthrax in my student-days was that of free (crucial) incisions as early as the correct diagnosis could be made, "the knife being passed freely through the tissues to the base of the inflammatory effusion, the object of which is to give room for the slough to separate and come away;" then poultice, and at the earliest moment dissect away all the slough as it formed. With such emphasis was this "crucial-incision treatment" dwelt upon by all teachers of this department of our art that it required a degree of temerity on the part of any one to deviate from this injunction.

Free incisions at times of necessity implied the useless infringement upon or passage through by your knife of tissues which were never to become involved in the destructive or breaking-down process; whose circulation and nerve-supply, as well as that of contiguous parts, were seriously jeopardized by this practice; also a loss of blood, which could not well be spared by some of the "run-down" patients.

This treatment, I have reason to believe, still generally prevails. The "caustic" treatment has some adherents, among the number an excellent and late writer, Mr. Bryant.

For several years past I have greatly departed from my early instructions upon this point; and, as I believe, not only thereby rendering the treatment less painful, but shortening the duration of the existence of the affection, and in addition saving tissues which under the old method would inevitably be destroyed.

Sidney Ringer (Handbook of Therapeutics) asserts, "Belladonna applied over abscesses and carbuncles reduces inflammation and allays pain." He advises its employment in any stage of inflammation, as "it will often arrest the progress of an abscess otherwise almost certain to maturate." Even when it fails to prevent suppuration "it will reduce inflammation, subdue much of the pain, and greatly limit the inevitable abscess."

As regards the use of poultices in these cases, my experience will not allow me to indorse their employment; for I am convinced they not only cause the formation of boils around the seat of the carbuncle, but produce an extension of the destruction of both integument and underlying tissues. I therefore never employ them.

When first seen, and recognized to be a carbuncle in its formative stage, make a small opening with a sharp-pointed bistoury in the center of the swollen and inflamed structures, just large enough to allow the easy introduction of the nozzle of a hypodermic syringe, which has been previously charged with a fifty-per-cent. solution of carbolic acid in oil or water, and after passing it a short distance into the central-forming slough, press the piston sufficiently to expel a drop or two of the contents of the syringe; retract and deflect the point of the syringe as you re-introduce, and repeat this until you have insinuated the solution into a considerable area of the interior of the commencing carbuncle. This done, with gentleness and patience rub into the overlying skin, upon and for a considerable distance around the forming anthrax, equal parts of extract belladonna and glycerine (Price's), finally applying a piece of lint well smeared with the same solution to the parts, strapping it in its proper place with gum-plaster, and over all this dressing a well-worn, soft silk handkerchief (folded). This external dressing should be repeated twice or oftener daily, with the double object of cleanliness and to get the supplying vessels impressed physiologically by the belladonna externally applied. As soon as the point of destruction of the integument is sufficiently large—or you are able to enlarge it by use of scissors or forceps and not cause great pain or hemorrhage—a piece of lint saturated in a fifty-per-cent. carbolized-oil solution should be gently but firmly introduced into the opening and, by spreading it out, be made to come in contact with the bottom of the inner surface of the carbuncle. This application causes at first some pain, but it will be short-lived, the patient soon appreciating the anesthetic effect of the carbolic acid. Upon the first piece of lint place a second piece (dry), and cover all with a third larger piece (three inches square), the inner surface of which has had a good coating of the belladonna-and-glycerin solution applied to it, securing the last with strips of plaster as before mentioned. At each succeeding dressing, as slough forms or breaks down into pus, remove carefully with forceps and scissors as much as you can, causing no bleeding, and as you approach the healthier parts beneath lessen the strength of carbolized oil or watery solution of acid you employ until you dilute to five grains to the ounce; finally, discarding altogether the acid solution, substitute for it either lukewarm water as a dressing, or, if indicated, a weak astringent solution. The carbolic acid has the effect of stimulating the circulation of the parts involved in the diseased action with which it is brought in contact, thus enabling them to repel this tendency to slough. It acts as a local anesthetic, together with the external application of the belladonna, removes to a great extent the usual necessity for the internal administration of sedatives to obtain sleep, and lessen pain. The glycerin and oil exclude the atmospherical air, thereby partly removing one necessary factor to the production of decomposition. The antiseptic and antiputrefactive quality of the acid reduces the danger of pyemic symptoms as a resulting complication to a minimum.

I would have no trouble in citing several cases which started out to all appearances for a six or eight weeks tour. Under the above mode of treatment, patiently carried out, sloughing and suppuration ceased, and healthy granulation began in from eight to twelve days.

As regards systemic treatment, if any of the functions are slothful, re-establish them. From the beginning give from one-twentieth to one-tenth grain doses of calcium sulphide, as advised by Ringer, continuing its use until healthy action takes place in the local trouble, and follow this when the symptoms of fever disappear with full doses of tinct. ferri. chlorid, *ter diem.* I can from experience indorse the assertion of Ringer, "In carbuncles the sulphides will generally be found serviceable, melting, as it were, the core into healthy pus, and so quickly expelling the dead and otherwise slow-separating tissue." They also break up the tendency to formation of boils or abscesses (cervical and others) in children of a scrofulous habit.—*Louisville Med. News.*

### THE WOMAN'S HOSPITAL.

An urgent appeal to the benevolent public has been made in a little pamphlet issued in behalf of the "Woman's's Hospital in the State of New York," which, after stating the origin, growth and usefulness of the institution, asks the financial aid now imperatively necessary to enable a continuance of the work which it long since began and has since faithfully continued to prosecute. The little pamphlet simply states the hospital's aim, and gives a few facts as to its accomplishments. But modestly as these last are explained the results are so great in benefits to suffering women that its appeal becomes at once a forcible and eloquent petition to the charitable, who should consider it both a duty and a privilege to supply the urgent pecuniary wants of the institution.

It needs no elaborate arguments to enable even the casual reader to understand how great a necessity existed for an extensive, well appointed woman's hospital—a place where the treatment of diseases peculiar to women could be pursued on so broad a plan that it should afford prompt and perfect relief to those afflicted with this peculiar class of affections. At the same time such a hospital opens a field for further and more intelligent study of medical and surgical practice to our physicians and surgeons, enabling them the more completely to comprehend and combat the ills to which our women are peculiarly exposed. It is universally admitted that the women of America are greatly prone to diseases of various and serious natures, and that the climate of the country, the physique of the people, the mode of their life, superinduce the development and inheritance of numerous grave maladies in the younger generations, which can be traced directly to the ill health and disordered state of the organs of the mothers of our land. It would be alarming indeed were it known how small is the proportion of healthy to sick young women in our midst to-day. To make the matter worse many of the most serious diseases to which women seem to be so naturally the heir are readily concealed in early life and are only discovered when the sensitive sufferer is compelled to seek prompt and efficient aid to save her life. Even then the oportunity for seeking relfef is exceedingly limited. The practice of that particular branch of the science and art of medicine and surgeury which is dedicated to the alleviation of woman's infirmities has been unable to make sufficient progress till late years, because of the lack of a proper field of observation for study and the lack of proper facilities for the accommodation of large numbers of patients, which can only be supplied by ample hospitals specially devoted to such cases. Consequently the scientific advance has been slow, and the opportunities afforded to patients in moderate circumstances to seek learned and skillful treatment have been meagre indeed. Physicians and surgeons have had to depend for knowledge on the study of isolated cases or the comparatively unprofitable reading of printed medical reports; and patients have had to seek, when they could afford it, such relief as could be had from the few specialists in their diseases, or, worse than ordinary death, submit to unintelligent treatment. The publicly unobserved growth of disease among American women has reached appalling proportions, and it was to meet this great and overshadowing evil that the Woman's Hospital was started in this city. No more touching trait exists in woman's character than the delicacy and modest sensitiveness which impels her to suffer in silence rather than to expose certain of her ills to the world, and it is a well known fact that hundreds of women have gone to the gates of the great city hospitals, driven there by the agony of their sufferings, and yet have turned back and determined to bear their bitter affliction rather than enter where men and women, though in different wards, are seeking common relief. "Were there a place where only women were near me on their sick beds," said one of these poor sensitive creatures, "I could enter, but I should feel as though I were exposing my sufferings to all the world were I to go to an ordinary hospital. I shrink from these hospitals, where in the next room or across the hall I know there are hundreds of men. I would rather feel and endure what is painful and distressing to me now, no matter how great the agony, than go to those hospitals. I suppose I am wrong, but I cannot help it, and I will go back home, I think." And she returned there to hide and nurse her disease, and of course to die.

### CLINICAL LECTURES ON CHILDREN'S DISEASES.

Our lesson to-day will be on the first duties of the medical man towards the young infant. These duties begin at the moment of its birth, while it is still connected with the placenta through the naval string or funis. This is a three-stranded cord usually between seventeen and twenty-four inches in length. The strands are blood vessels —one vein and two arteries. The single vein is as large as both the arteries. It has carried blood from the placenta to the child. The arteries, though smaller, are much longer than the vein, and often wind spirally around it. They have conveyed impure blood from the fœtus to the placenta. I suppose they were first called arteries because they pulsate. The funis also contains a gelatinous material, the gelatine of Wharton, light in color, that surrounds the blood-vessels and holds them in place. The quantity of this substance is variable ; hence there are considerable differences as regards size in different cords. A cord may be as small as the stem of a clay-pipe or as large as your middle finger. The average size is about that of the little finger. The funis has also a membranous external covering derived from the amnion.

Now, if the infant is breathing properly, its blood is being renovated at its own lungs, and both the cord and placenta are henceforth useless. The first duty of the physician towards the child is to relieve it of this encumbrance. This he does by tying and dividing the cord.

The formation of the ligature, and the place and manner of its application, are matters of some moment. A tape or ribbon, or any small string, will answer to tie with ; but I prefer to have it done in this way : From six to ten strands of white spool cotton of medium size, No. 24 to 40, from ten to twelve inches long, are tied together at either end. These form a ligature so large that there is no danger of cutting through the funis, however tightly it is drawn, while it is an effectual guard against hemorrhage, since each individual strand exercises a certain degree of constriction independently of the others. It is, in fact, several ligatures in one. I make two of these strings. The first is applied about an inch and a half from the abdomen. This distance is selected in order to avoid a possible umbilical hernia. Pass the ligature two or three times around the funis, draw it tightly, tie it and cut the ends short. Apply the second string two or three inches to the placental side of the first, previously stripping the cord with the fingers to expel the blood from the space between them. The object of this second ligature is to prevent the escape of blood from the placenta over the bed and clothing. Being of little importance as compared with the other, less pains need be taken with it. The next step is to divide the cord. The section should be made about half an inch below the first ligature. The best instrument is the blunt-pointed scissors, though I have done it with my pocket-knife. The section should be made without haste and in a good light. Through carelessness an infant's finger has sometimes been mutilated by the shears. And be sure to make the division at the right point. I knew a good physician, after a tedious labor that had fatigued him, to cut the cord between the umbilicus and the first ligature. The infant nearly bled to death before the mistake was discovered.

The operation completed, you may place the child in the blanket that the nurse has provided. It is now to be washed, clothed and fed. You will not, of course, be expected to do these things yourselves, but you should know about them, for you will have many occasions for giving counsel concerning them.

The initial cleansing and dressing should take place in a warm room. Too little thought is given to this point, and infants often become seriously, if not fatally, chilled at this time. If you remember that the babe has just left a dwelling where its fœtal life has been passed in a temperature of almost 100 deg., you will not allow it to be exposed in an atmosphere much below 90 deg. before it is dressed. The water for washing should be from 92 deg. to 97 deg.

Frequently the infant is covered over with a whitish coating—the *vernix caseosa* or " "cheesy varnish." It is composed of fatty matter from the sabaceous follicles and exfoliated epithelium. This is not easy of removal by an ordinary washing. But first besmear with some greasy substance as lard, olive oil, vaseline or butter, or the yolk of an egg, then the removal is readily effected by soap and water.

In dressing the child, the first thing to be attended to is the proper covering of the stump of the umbilical cord. I do it in this way—though, of course, the way is not original with me :

From the old linen cloth usually provided for the purpose, I shape a piece four and a half by six inches. In the centre I make a foramen large enough to give easy admittance to the stump, which I then pass through it. I then spread the cloth lengthwise along the child's abdomen, and, turning the cord upward, lay it down upon it. The lower half of the cloth is then folded over and laid upon the cord. The nurse should now apply a binder smoothly, but not too tightly over this, and the dressing is complete. It is an old custom to grease the cloth with suet. I think it better not to do it, as it tends to hinder the drying of the stump. It is at best a useless practice.

As the power of the newly-born child to evolve heat is feeble, the best material for under clothing is flannel—a non-conductor. The dress should be loose enough to allow of free movement. Tapes are preferable to pins for fastening the garments.

The child being washed and dressed, is next to be fed. It is well to have the child applied to the breast after allowing the mother some rest—say from two to four hours. The secretion at this time is not exactly milk, but a thick, yellowish, fatty fluid, known as *colostrum*. It is nutritious and laxative, and nature has specially prepared it for the child's first food. It is too much the custom to keep the child from the breast and to feed with artificial mixtures until after the third day.

If the mammary secretion should be insufficient in amount for the first two or three days, I direct the deficiency to be made good by giving cow's milk with one-fourth part of water and a little sugar.

But this brings us to the great subject of infant dietetics, to which we must devote a full hour at another time.—*W. T. Plant, M. D., in Obstetric Gazette.*

### ELECTRICITY IN THE TREATMENT OF EXOPTHALMIC GOITRE.

In the *New Orleans Medical Journal* for June, 1881, Dr. A. D. Rockwell, Electro-therapeutist to the New York State Woman's Hospital, alludes to eight cases of exopthalmic goitre previously recorded by him as having been treated with electricity--three ending with recovery, and one in approximate recovery, and gives the history of an additional case in which the result was favorable. It would be impossible, he thinks, to obtain similar results in a number of cases by any one method of electrical treatment. In some cases localized galvanization by the ordinary method may prove efficacious. This method may thus be described: Place the cathode over the cilio-spinal center, above the seventh cervical vertebra, and the anode in the auricular-maxillary fossa, gradually drawing the latter (after a few moments of stabile treatment) along the inner border of the sterno-cleido-mastoideous muscle, to its lower extremity. The second step in this process consists in removing the anode to the position occupied by the cathode, and placing the latter over the solar plexus, using for a few moments longer a greatly increased strength of current. In other cases currents alternately increased and diminished may prove most effective. The general application of the faradaic current sometimes proves an important factor in the method of treatment. It is not very difficult to believe, he remarks, nor to understand why, general faradization is so effective in lowering a pulse that is rapid as a result of nervous excitement, and in increasing its strength when it is both rapid and weak through nervous exhaustion. It is more difficult to explain why this result is so pleasantly attainable in cases of exopthalmic goitre in which the galvanic current, after benefiting up to a certain point, fails to do more. The faradaic certainly does not affect the sympathetic so directly and powerfully as the galvanic current does, and we are obliged, for an explanation, to refer to its well known superior tonic properties, and to the fact that the complete and thorough excitation of the cutaneous nerves by general faradization is followed by a greater and more desirable reflex influence. In a case of over thirty years' standing, which the author recently treated, but in which he failed to cause any appreciable reduction in size, this power of one current to supplement the action of the other was well illustrated. The pulse of the patient was constantly at or above 115. The action of the galvanic current reduced it to 105, but failed to do more than this after considerable effort. General faradization was then attempted, with the result of affecting within a week a further and seemingly permanent reduction of twelve beats. At the same time the patient's general condition was much improved.

### ODOR MORTIS.

In the summer of 1875, Dr. A. B. Isham read apaper before the Cincinnati Academy of Medicine, based upon observations made in hospital and private practice since 1863, concerning a peculiar odor noticed at the dead bedside. He now reports two new instances in the *Am. Jour. of Med. Science* for April, 1881. In one of these cases he distinguished the odor thirty-three hours before death, in the other only one and one-half hours before, at which time he was called to the patient. He describes the odor as moschiferous, yet possessing an appreciable yet indescribable difference from the odor of musk, the impression upon the olfactory organs being more delicate, more subtile. The odor was familiar to convalescents of the Stanton Hospital, and it was not uncommon to hear one say, "some one is dying, for I can smell him," but the doctor says he has been able to find only the following quotation, taken from the *British Med. Journal*, referring to anything analogous in literature: "I hazard the idea that the ammoniacal odor emanating from the living body, so strong on opening the large cavities, and so striking on receiving some of the blood out of the vessels, arteries as well as veins, into the hand, were all due to the same condition of this fluid; the actual presence of ammoniacal salts, one of the surest proofs of the putrescent condition of the vital fluid; in fact, to speak paradoxically, of the existence of death during life." The above was written by Dr. Badgley, of Montreal, in a report on Irish emigrant fever. The source of the smell which he indicates agrees with Dr. Isham's theory. Robiquet holds that the odor of musk originates in the decomposition of the volatile oil by the ammonia which enters into the composition of the musk. Dr. I. thinks that the ammonia of the blood is being set free as death approaches and acts upon the volatile oil of the blood. Whatever may be the explanation, the facts, if of sufficiently frequent occurrence, may prove very valuable aid in diagnosis.—*Detroit Lancet.*

### THE BERNHARDT CRAZE.

The *Medical Record* went into ecstacies (?) over her histrionic deaths; the *Canadian Journal of Medical Sciences* calls her the "divine Sarah," and in admiration of her attenuated form quotes from the Chicago *Medical Journal and Examiner* which says that the principal interest in a medical point of view was the fact that "she was so thin that when she took a pill she looked as if she was pregnant."—*Michigan Medical News.*

